# The Effect of Phytocannabinoids and Endocannabinoids on Nrf2 Activity in the Central Nervous System and Periphery

**DOI:** 10.3390/neurolint16040057

**Published:** 2024-07-18

**Authors:** Pietro Marini, Mauro Maccarrone, Luciano Saso, Paolo Tucci

**Affiliations:** 1 Institute of Education in Healthcare and Medical Sciences, Foresterhill Campus, University of Aberdeen, Aberdeen AB25 2ZD, UK; 2 Department of Biotechnological and Applied Clinical Sciences, University of L’Aquila, Via Vetoio snc, Coppito, 67100 L’Aquila, Italy; 3 European Center for Brain Research (CERC), Santa Lucia Foundation IRCCS, Via del Fosso di Fiorano 64, 00143 Rome, Italy; 4 Department of Physiology and Pharmacology “Vittorio Erspamer”, Sapienza University of Rome, 00185 Rome, Italy; 5 Department of Clinical and Experimental Medicine, University of Foggia, 71122 Foggia, Italy

**Keywords:** Nrf2, phytocannabinoids, endocannabinoids, oxidative stress

## Abstract

The relationship between nuclear factor erythroid 2-related factor 2 (Nrf2) and phytocannabinoids/endocannabinoids (pCBs/eCBs) has been investigated in a variety of models of peripheral illnesses, with little clarification on their interaction within the central nervous system (CNS). In this context, evidence suggests that the Nrf2-pCBs/eCBS interaction is relevant in modulating peroxidation processes and the antioxidant system. Nrf2, one of the regulators of cellular redox homeostasis, appears to have a protective role toward damaging insults to neurons and glia by enhancing those genes involved in the regulation of homeostatic processes. Specifically in microglia and macroglia cells, Nrf2 can be activated, and its signaling pathway modulated, by both pCBs and eCBs. However, the precise effects of pCBs and eCBs on the Nrf2 signaling pathway are not completely elucidated yet, making their potential clinical employment still not fully understood.

## 1. Introduction

The scientific community and general public are still debating whether the well-known beneficial effects of cannabis-based drugs could justify their clinical employment, despite the potential risks associated with the use of this class of compounds. Recently, after a revision of the scientific literature conducted by the Expert Committee on Drug Dependence (ECDD), an independent scientific advisory board to the World Health Organization (WHO), the positive effects of cannabis were clarified in comparison to the negative ones. As a result, the Commission on Narcotic Drugs (CND) of the United Nations removed cannabis and its derivatives from Schedule IV of the 1961 Single Convention on Narcotic Drugs in 2020 [1] and reclassified these substances as Schedule III. Although still considering cannabis and its derivates potentially harmful, cannabis’ medicinal and therapeutic potentials have been recognized. In 2024, the United States Drug Enforcement Administration has consented to reclassify cannabis as a lower risk drug [2]. The trichomes, specialized inflorescence structures of female cannabis plant, produce a family of terpenophenolic substances, called phytocannabinoids (pCBs), representing more than 110 of the nearly 500 compounds (terpenoids, flavonoids, sterols, and other non-pCB substances) found in all cannabis subspecies (*Cannabis sativa*, *Cannabis indica,* and *Cannabis rudelalis*) [3,4,5]. Among all pCBs, three molecules have garnered noteworthy attention: (-)-Δ^9^-tetrahydrocannabinol (THC), (-)-cannabidiol, (CBD) and cannabigerol (CBG). Initially, it was believed that THC’s effect, because of its lipophilic properties, was caused by a general disruption of cell membranes. Thereafter, it was proved that the pharmacological properties of THC were to be ascribed to its stereoselectivity, since synthetic (+)-Δ^9^-tetrahydrocannabinol enantiomer did not show the same effects, hence suggesting the existence of a putative receptor. Indeed, cannabinoid receptors 1 and 2 (CB_1_R and CB_2_R) were subsequently identified, and numerous ligands were synthesized, laying the foundations for extensive research on the potential use of cannabis-related substances in medicine [6]. Nowadays, many different CB_1_ and CB_2_ receptors ligands are available either directly extracted from the cannabis plant (pCBs) or manufactured in the laboratory (synthetic cannabinoids), with pharmacological characteristics such as agonists, partial agonists, antagonists, and inverse agonists [7]. Moreover, the molecular mechanism of action of pCBs on CB_1_R and CB_2_R, as well as the ability of some pCBs to bind non-CB_1_/CB_2_ receptors (i.e., GPR55, GPR18, 5-HT_3_, 5-HT_1A_, TRPV1, GPR119, GlyRs, and PPARs), have been extensively elucidated [6]. For example, THC behaves as an agonist while CBD as a negative allosteric modulator at CB_1_R [8]. The pCBs compounds are currently prescribed for the treatment of some pathological conditions. Sativex^®^, a 1:1 THC/CBD formulation available as mouth spray, has been approved for the treatment of multiple sclerosis symptoms, such as spasticity, neuropathic pain, and overactive bladder [9], while a CBD-based preparation has been approved for the treatment of the Lennox–Gastaut and Dravet epileptic syndromes, two types of childhood-onset epilepsies [10]. Furthermore, synthetic CB_1_R agonists are used in various medical conditions; for example, dronabinol and nabilone are prescribed to increase appetite or to reduce vomiting in patients receiving chemotherapy [9]. Unfortunately, a synthetic CB_1_R inverse agonist, Rimonabant, initially approved as an anti-obesity medication, was withdrawn globally in 2008 due to the serious psychiatric side effects reported [11]. The discovery of CB_1_R and CB_2_R prompted intense research into the identification of potential endogenous receptor ligands, with anandamide (*N*-arachidonoylethanolamine; AEA) [12] and 2-arachidonoylglycerol (2-AG) being identified [13,14] and collectively termed as endocannabinoids (eCBs). Subsequently, the enzymes responsible for eCBs synthesis and degradation were also discovered, leading to the complete identification and description of the termed endocannabinoid system. The possibility to modulate eCBs production by targeting metabolic enzymes has represented another potential clinical application. The inhibition of the fatty acid amide hydrolase (FAAH), the enzyme responsible for AEA degradation, was initially considered as beneficial for those conditions where reduced levels of eCBs had impairing effects. Although several studies demonstrated the potential benefits of eCB modulation using FAAH inhibitors (i.e., PF04457845, a highly selective and clinically tested FAAH inhibitor), the clinical application of such inhibitors was shelved following the tragic phase I clinical trial in which the purported FAAH inhibitor BIA 10-2474 was tested. The drug caused severe neurologic side effects leading to the death of a healthy volunteer [15]. Further investigations clearly demonstrated how the drug was able to inhibit not only FAAH, but also several other lipases, producing substantial alterations of lipid networks in human cortical neurons, highlighting how promiscuous lipase inhibitors, rather than authentic FAAH inhibitors, may cause severe metabolic dysregulation and neurotoxicity [16].

## 2. Cannabinoids and Endocannabinoids as Antioxidants

**Figure 1:**
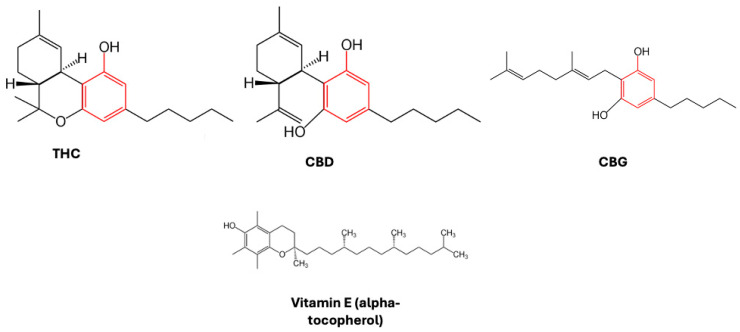
Chemical structure of pCBs reported. The phenolic group is highlighted in red. As a comparison, the structure of a traditional antioxidant, vitamin E (alpha-tocopherol), is also shown.

By looking at the chemical structures of THC, CBD, and other pCBs, it is evident that they all contain a phenolic group and double bonds (Figure 1). 

The phenolic group is prone to oxidation, as proven by a reddish-purple hue that appeared when methanolic KOH was applied to hashish fiber, with hydroxyquinone (cannabidiolquinone) being produced, for example, by the aerobic oxidation of cannabidiol [17]. Cannabidiolquinone’s distinct reddish color is a result of conjugated double bonds in its molecular structure, with delocalized electrons produced by alternating single and double bonds able to absorb visible light. When it comes to CBD-chinol, the conjugated double bond arrangement causes light to be absorbed in the blue-green portion of the spectrum; therefore, red light is reflected, giving the characteristic coloration (reddish-purple) of hashish fibers. In laboratory tests, the color shift of CBD to its oxidized form, CBD-chinol, is a visual indicator for the presence of cannabinoids in the specimens and that oxidation processes are taking place [18]. In this context, two mechanisms have been proposed to be responsible for the antioxidant effect of CBD. In the first one, an electron is transferred from the phenolic group of CBD to a free radical, whereas in the second one, a hydrogen atom is removed by a free radical from CBD. In both cases, to avoid chain radical reactions, the produced radicals need to be stable [19]. These chemical characteristics of cannabinoids make these compounds good antioxidants, able to scavenge free radicals, to protect against oxidation processes, and to reduce metal ions. Although the antioxidant activity is not the same for each cannabinoid compound, the activity is generally comparable to that of vitamin E [20,21]. In particular, since both THC- and CBD-cation-free radicals exhibit a number of resonance structures, with unpaired electrons primarily distributed on both the ether and alkyl moieties, as well as on the benzene ring, these compounds may potentially have antioxidant properties [19,21]. Indeed, in a landmark study conducted in rat cortical neurons, both CBD and THC were able to decrease glutamate toxicity and reactive oxygen species (ROS)-induced cell death [22]. These effects were proposed to be CBR-independent, since the neuroprotection caused by both compounds was still noticeable when CB_1_R were antagonized.

## 3. The Antioxidant Mechanisms

The role of pro-oxidants in the etiopathogenesis and development of various diseases has triggered an interest toward the antioxidant properties of pCBs. Generally, cellular metabolized ROS species are classified as pro-oxidants with a bell-shaped concentration profile. At lower concentrations, they maintain physiological cell processes, whereas at higher concentrations they cause harmful alterations to DNA, lipids, and proteins (for a detailed review, see [23]). 

The transition from a lower to a higher concentration of cellular metabolized ROS species results in “oxidative stress”, a well-known factor involved in a variety of pathological conditions (i.e., neurological disorders such as Parkinson’s disease, Alzheimer’s disease, motor neuron disease [24]), with hydrogen peroxide, hydroxyl radicals, peroxyl radicals, hydroperoxyl radicals, and hypochlorous acid representing the primary endogenous oxidant species mainly contributing to ROS. On the other hand, the negative effects that ROS have on cellular metabolism are counteracted by an endogenous and integrated intracellular antioxidant mechanism composed of enzymatic and non-enzymatic antioxidants systems [25].

Specifically, superoxide dismutase (SOD), catalase (CAT), glutathione peroxidase (GTPx), thioredoxin (TRX), peroxidase, peroxiredoxin (PRX), NAD(P)H dehydrogenase (quinone)-1 (NQO-1), and heme oxygenase-1 (HO-1) are all examples of enzymatic systems, while β-carotene, vitamins A, E and C, as well as glutathione (GSH), are all examples of non-enzymatic systems. In this context, it is important to note that the genes coding for the enzymes responsible for the cellular control of GSH levels, such as glutathione reductase (GR), γ-glutamyl cysteine synthetase (GCL), and γ-glutamine cysteine synthase (GCS), are all regulated by the nuclear factor erythroid 2-related factor 2 (Nrf2), which is also involved in the genetic regulation of both NQO-1 and HO-1 [26]. 

By binding to the antioxidant response element (ARE) located in the promoter region of every detoxifying gene, the transcription factor Nrf2 is widely recognized as one of the regulators of cellular redox homeostasis, antioxidant defense, and detoxification [27]. 

## 4. Nrf2 Pathway

In homeostatic conditions within the cell cytoplasm, Nrf2 is normally bound to the Keap1-Cul3-RBX1 complex, composed of RING-box protein 1 (RBX1), Cullin-based (Cul3) E3 ligase, and Kelch-like ECH-associated protein 1 (Keap1). The complex, when bound to the Neh2 domain of Nrf2, facilitates its ubiquitination and proteasomal degradation [28,29,30,31,32,33,34,35]. 

In particular, the oxidative modification of cysteine residues in Keap1, induced by exposure to stressors such as electrophiles or excessive production of ROS, causes the dissociation of Nrf2 from the Keap1-Cul3-RBX1 complex and subsequently its migration into the nucleus. Here, heterodimerization with the small Maf protein occurs, leading to gene activation through binding to the ARE promoter region [35,36]. Other evidence suggests that Nrf2 activity can also be regulated by the beta-transducin repeats-containing protein (β-TrCP) and the Skp1-Cul1-Rbx1 ubiquitin ligase complex [26], while its proteasomal degradation could be initiated by the glycogen synthase kinase-3 (GSK3) through phosphorylation processes [26]. Furthermore, evidence of crosstalk between Nrf2 and the nuclear factor kappa-light-chain-enhancer of activated B cells (NF-κB), this latter itself a redox-sensitive transcription factor, has been reported [37]. In particular, proposed mechanisms suggest that both transcriptional factors compete for the CREB binding protein (CBP) site in the nucleus, with NF-κB recruiting histone deacetylase 3 (HDAC3) and therefore inhibiting Nrf2 ARE-dependent gene expression. In contrast, Nrf2 could be indirectly activated by anti-inflammatory compounds that suppress NF-κB activity and vice versa, NF-κB could be indirectly activated by Nrf2 inhibitors [37]. 

Finally, in the brain, Nrf2 exerts its anti-inflammatory properties by reducing neuroinflammation brought on by damaging stimuli associated with neurodegenerative diseases. In this context, Nrf2 indirectly contribute to the formation of the multimeric protein complex, inflammasome NLRP3 [38], thus playing a critical central defensive role against oxidative stress in brain pathophysiology, such as gliosis, proteinopathy, oxidative and inflammatory stress [39]. 

## 5. Effects of Phytocannabinoids and Endocannabinoids on Nrf2 Activity in the Central Nervous System (CNS) 

It is not surprising that the relationship between pCBs/eCBs and Nrf2 has been first studied in brain cell models, given the well-known effect cannabinoids have on the CNS. The increased turnover occurring in lipid peroxidation processes and the central role played by the antioxidant system in the brain poses valid reasons for further investigation into the interaction between pCBs/eCBs and Nrf2. It has been demonstrated that THC causes glioma cells to undergo apoptosis [40], with a concomitant increase in intracellular ROS, as well as a decrease in GSH. In neonatal mice (post-natal day 10) exposed to a single dose of THC: (*i*) an increase in the Nrf2/Keap1 ratio was observed in both parietal cortex and hippocampus; (*ii*) increased levels of the apoptosis regulator BAX transcript were observed in the frontal cortex; (*iii*) increased levels of CB_1_R transcript were observed in the parietal cortex [41]. 

The Nrf2 and cannabinoid signaling pathways have been demonstrated to closely interact with each other in various models of neuropathic pain. For example, in male type 2 diabetic mice (BKS.Cg-m+/+Leprdb/J; db/db), the activation of the antioxidant Nrf2/HO-1 pathway amplified the antiallodynic effects of CB_2_R agonists JWH-015 and JWH-133 [42].

In addition, neuropathic mice treated with a combination of CBD and Δ^9^-tetrahydrocannabivarin (THCV) exhibited increased expressions of Nrf2, HO-1, and catalase in dorsal root ganglions. 

It is noteworthy that the pCB THCV is a neutral, non-psychoactive CB_1_R antagonist/inverse agonist which, depending on the dose, can also behave as either an agonist or an antagonist at CB_2_R [43]. 

The combination of these pCBs resulted in a boost in their antioxidant potential, by lowering neuropathic pain and improving mitochondrial function, primarily acting on the activation of the AMPK-Nrf2-mitochondrial transcription factor A (TFAM) signaling cascade [44]. When phosphorylated, AMPK increases mitochondriogenesis and respiratory capacity through TFAM activation. Upon its activation, TFAM binds to the mitochondrial genome and regulates the transcription of mitochondrial subunit complexes, thereby decreasing the mitochondrial functional deficiencies observed, for example, in animal and cell culture models of neuropathic pain and diabetes [44,45].

Interesting studies were conducted on CB_2_Rs, whose activation does not result in psychoactive effects, to further elucidate their involvement in the regulation of Nrf2 within the CNS. These studies were conducted by using the CB_2_R selective agonist, β-caryophyllene (BCP) [46], a naturally occurring bicyclic sesquiterpene, classified as pCB, found in the essential oils of various species, such as *Cannabis sativa*, *Piper nigrum*, and *Cinnamomum* spp. [47].

When tested in the BV-2 cell line, BCP was able to regulate cellular antioxidant responses, primarily by preventing ROS production, restoring mitochondrial membrane potential, and protecting microglial cells from glutamate cytotoxicity. Moreover, BCP was also able to promote Nrf2 nuclear translocation, thereby enhancing the astrocytes’ cellular GSH antioxidant system [48]. Particularly, rat BV-2 cells were frequently used as an in vitro model to investigate the function of the endocannabinoid system in microglia, since this cell line, endocannabinoids, metabolic enzymes (i.e., FAAH), and CB_2_R are all constitutively expressed [49,50,51]. 

In oligodendrocytes (OLN-93), cells that also naturally express CB_2_Rs, BCP was able to prevent LPS-induced cytotoxicity, as well as ROS, TNF-α, and nitric oxide production. The protective effect of BCP was mediated by CB_2_R stimulation through the regulation of the Nrf2/HO-1/antioxidant axis and PPAR-γ pathways [52]. 

Similarly, classical pCBs were studied in experimental models of LPS-stimulated toxicity. For example, CBG, a non-psychoactive pCB, has shown protective effects on both RAW 264.7 (macrophages) and NSC-34 (motor neuron) cell lines, suggesting its possible application in the treatment of neurodegeneration, as well as in other pathological conditions, where oxidative stress and neuroinflammation are major factors, such as Huntington disease, Parkinson disease, multiple sclerosis [53]. Indeed, in an experimental model of neuroinflammation, pretreatment with CBG was observed to lower nitrotyrosine, SOD1, and inducible nitric oxide synthases (iNOS) protein levels, while increasing Nrf-2 levels and preventing apoptosis [54], with similar effects also observed in the presence of CBG-CBD co-administration [55].

Furthermore, in a mouse experimental model of chronic autoimmune encephalomyelitis (EAE) induced by myelin oligodendrocyte glycoprotein peptide 35–55 (MOG35-55), CBD administration was observed to increase the expression of Nrf2 target genes, metallothioneins (Mt1 and Mt2), and Hmox-1 [56]. 

In BV-2 cells, CBD administration was able to stimulate those genes known to be involved in the regulation of inflammation and stress response, primarily through the Nrf2/Hmox1 axis and the ARE-Nrf2/ATF4 system [57,58]. In LPS-stimulated BV-2 cells, a particular repertoire of miRNAs was regulated by cannabinoids with TLR, Nrf2, and Notch crosstalk signaling, reported as responsible for the regulation of altered miRNAs of genes involved in the immune response, cell cycle regulation, cellular stress, and redox homeostasis [59].

The specific role played by CB_2_R in the above-mentioned mechanisms was further elucidated in other experimental models, such as primary cultures of rat microglia cells, where the administration of the synthetic CB_2_R selective agonist JWH133 was able to promote PI3K/Akt activation and therefore facilitate the nuclear translocation of Nrf2 [60].

Furthermore, in primary microglial cultures derived from Abcd1-null mice and from patients with X-linked adrenoleukodystrophy (X-ALD), the administration of JWH133 induced the Nrf2 antioxidant pathway and inhibited the ROS production elicited by excess very long-chain fatty acids [61]. 

The CNR2 gene encoding for CB_2_R contains a putative ARE motif that exhibits strong similarities to the consensus ARE sequence found in the Nrf2 promoter region [62]. In both hippocampal HT-22 cells and primary neonatal neurons from the mouse cortex, evidence suggested that Nrf2 was unable to control CB_2_R expression, while in microglial cells, the expression of the receptor was found to be Nrf2-dependent, suggesting that CNR2 gene activation could be mediated by different transcriptional factors in different cell types [63].

Collectively, the effects exerted by exogenous CB_2_R agonists on the Nrf2 pathway suggest a possible physiological role of eCBs in modulating/regulating the transcript activity.

For example, in HT-22 cells, those eCBs containing a phenolic moiety, such as N-acyl dopamines or N-arachidonoyl 5-HT, have shown an ability to exert antioxidant or anti-inflammatory actions through the activation of the Nrf2-mediated antioxidant response. Similar effects were also shown by the pCBs THC and CBD [64]. In a neurotoxic model of primary hippocampal hyperglycemic neurons and oligomeric amyloid β peptide (Aβ1-42), the involvement of eCBs in the regulation of Nrf2 activity has been demonstrated [65]. In these models, eCBs AEA and 2-AG, as well as the synthetic cannabinoids CP 55–940 and WIN 55,212–2, all reduced the assessed toxic endpoints. However, the strongest effect was observed in presence of URB597, an inhibitor of the FAAH enzyme responsible for AEA hydrolysis. This compound was the only one able to prevent the toxicity caused by high glucose and amyloid without raising Nrf2 and CREB phosphorylation [65]. The models used to evaluate the effects of pCBs and eCBs on central Nrf2 in vitro and in vivo are compiled in Table 1.

## 6. The Effects of Phytocannabinoids and Endocannabinoids on Nrf2 Activity in the Periphery

Outside of the CNS, the cannabinoid-mediated activation of Nrf2 was demonstrated in various models of peripheral illnesses. In experiments using SV-HUC1 cells (TNFα-stimulated normal human urothelial cells), an in vitro model of bladder pain syndrome and interstitial cystitis, administration of CBD was able to enhance the redox-sensitive transcription factor Nrf2 along with the expression of both the antioxidant enzymes, SOD 1 and 2 and the HO-1, potentially through the activation of the PPARγ receptor and attenuation of the NF-kB pathway [66]. Similarly, in mouse hepatocytes and L-02 cells exposed to α-amanitin, the lethal toxin of *Amanita muscaria*, CBD was able to upregulate either Nrf2 and both HO-1 and NADPH-Quinone Oxidoreductase1 (NQO1) antioxidant enzymes levels, thus attenuating the oxidative stress and apoptosis induced by the toxin [67].

Furthermore, CBD was found to reduce the severity of 5-fluorouracil-induced oral mucositis in mice and human oral keratinocytes by upregulating the expression level of antioxidant enzymes, such as HO-1 and NQO1, as well as by increasing the expression of Nrf2 and its nuclear translocation, all effects being concomitant with a decrease in Keap1 activation (Nrf2 suppressor). Both the Nrf2 inhibitor ML385 and Nrf2-siRNA transfection neutralized the protective effects of CBD, indicating the direct interaction between this cannabinoid compound and Nrf2 activation [68].

In many studies conducted on epidermal skin cell keratinocytes exposed to UVA and UVB radiation, CBD was observed to affect the interaction between Nrf2-NFκB transcription factors, promoting the activation of the former and suppressing the activation of the latter [69]. Further evidence of CBD ability to induce the expression of selected Nrf2 target genes was provided by experiments conducted on primary and immortalized human keratinocytes (HaCaT cell line). Interestingly, although CBD was significantly less effective than sulphoraphane (SFN) (a metabolite of glucoraphanin found in *Brassica oleracea* known to activate Nrf2 [70]) at inducing the expression of the main Nrf2 target genes aldo-ketoreductases AKR1B10 and AKR1C1, the compound was instead equally effective, or even more effective, at inducing the expression of specific Nrf2 subset target genes: HMOX1, glutamate-cysteine ligase catalytic subunit (GCLC), and p62 [71,72].

Moreover, in both keratinocytes and epidermal equivalents, CBD enhanced the expression of filaggrin, involucrin, Nrf2, and NQO1, as well as increased the expression of aryl hydrocarbon receptor target genes such as CYP1A1 and aryl hydrocarbon receptor repressor [73]. In experiments on skin rats exposed to UV light, topical application of CBD for four weeks induced a lower expression of SOD and a consequent decrease in the UV-enhanced levels of Nrf2, leading to an impaired cytoprotective effect in keratinocytes [74]. By considering that either activation or accumulation of Nrf2 could favor a suitable environment for the growing and proliferation of neoplastic, chemo- and radio-resistant cells, compounds such as CBD, with their ability to reduce Nrf2 levels, may provide a cellular protective effect [75].

In mice with imiquimod-induced experimental psoriasiform skin lesions, a significant improvement (decrease in both plaque and epidermal thickness) was observed upon CB_2_R activation. When compared to the control groups, the treated animals showed higher levels of Nrf2 and HO-1 protein expression, suggesting the involvement of the NF-κB and Keap1/Nrf2 pathways in CB_2_R’s downstream signaling [76]. 

It has been demonstrated that CB_2_R is involved in damaging H_2_O_2_-induced C2C12 myoblasts in vitro. In fact, pretreatment with the CB_2_R agonist AM1241 prevented the H_2_O_2_-induced reduction in C2C12 cell viability, reduced reactive oxygen species generation, and increased the expression of Nrf2 and its nuclear translocation [77]. 

This mechanism is further supported by evidence obtained in Nrf2 knockout mice, where degenerative oxidative damage, myogenesis, and skeletal muscle deterioration were observed upon AM1241 administration. Similarly, in C2C12 cells, the administration of the compound impaired differentiation [77]. The protective role of CB_2_R in its ability to promote skeletal muscle repair following ischemia-reperfusion injury has also been demonstrated [78], suggesting a significant role of CB_2_R in the musculoskeletal system. For example, hFOB 1.19 osteoblasts treated with the CB_2_R agonist HU308 showed decreased p62 expression and Nrf2 degradation [79]. In RAW 264.7 macrophage cells, osteoclast differentiation was stimulated by AM1241 and suppressed by the CB_2_R-selective antagonist, AM630. Although the expression of both HO-1 and Nrf2 was increased by AM1241 and AM630, only the CB_2_R selective antagonist was able to effectively activate the HO-1/Nrf2 pathway and to promote a decrease in osteoclast differentiation [80]. 

In contrast, in an experimental model of an infarcted heart, the administration of AM1241 improved the adverse oxidative stress and inflammation milieu through the upregulation of the fosfoinositide 3-chinasi (PI3K)/protein kinase B (Akt)/Nrf2 signal pathway [81], as well as reduced myocardial interstitial fibrosis through the Nrf2-mediated down-regulation of the transforming growth factor beta 1 (TGF-β1)/Smad3 pathway [82]. 

In the macrophage RAW264.7 cell line, administration of JWH-133 was able to polarize M1 macrophages via activation of the Nrf2/HO-1 pathway and the effect was reduced by the HO-1 inhibitor, Sn(IV) protoporphyrin IX dichloride [83].

In C57BL/6 male mice trained in rigorous exercise, CBD administration showed a protective effect against myocardial injury via the Keap1/Nrf2/HO-1 pathway activation, causing the down-regulation of Keap1 protein expression, increasing Nrf2 translocation into the nucleus, and therefore promoting the expression of the antioxidant protein HO-1 [84]. The strong CBD interaction with Keap1/Nrf2/HO-1 signaling pathway in myocardial injury induced by intensive exercise was further supported by molecular docking experiments [84].

Several studies using cancer cell models have also highlighted the involvement of eCBs in Nrf2 function and regulation. In breast cancer models, such as MCF-7 and MDA-MB-231 cell lines, independently of CBRs activation, administration of AEA or the suppression of its hydrolase FAAH, both activated Nrf2 and consequently induced HO-1 [85]. These findings suggest that in breast cancer cell survival, the Nrf2-HO-1 pathway may be directly activated by AEA in a non-receptor-mediated manner. The compound also appears able to target/modulate the function of endothelial cells, tumor macrophages, and tumor fibroblasts, all elements present in the microenvironment niche surrounding tumor cells in vivo [85]. This suggests a possible role played by this endocannabinoid in the Nrf2 pathway activation, as well as its potential involvement in chemotherapy resistance. Indeed, the role of cannabinoids on Nrf2-related factors has been recently reviewed in the context of cancer prevention and treatment [86], with an amount of evidence reporting the beneficial and protective role provided by the modulation of AEA/HO-1 signaling as a coadjutant pharmacological approach to reduce radio resistance and chemoresistance [85]. 

It is noteworthy that in HaCaT cells, hexocannabitriol, a hydroxylated CBD analogue isolated from hemp threshing residues, was recently shown to activate the Nrf2 pathway in a ROS-independent way, most likely as a result of direct Nrf2 stabilization [87]. Table 2 summarizes the models utilized to assess the effects of pCBs and eCBs on peripheral Nrf2 both in vitro and in vivo.

## 7. Conclusions

**Figure 2:**
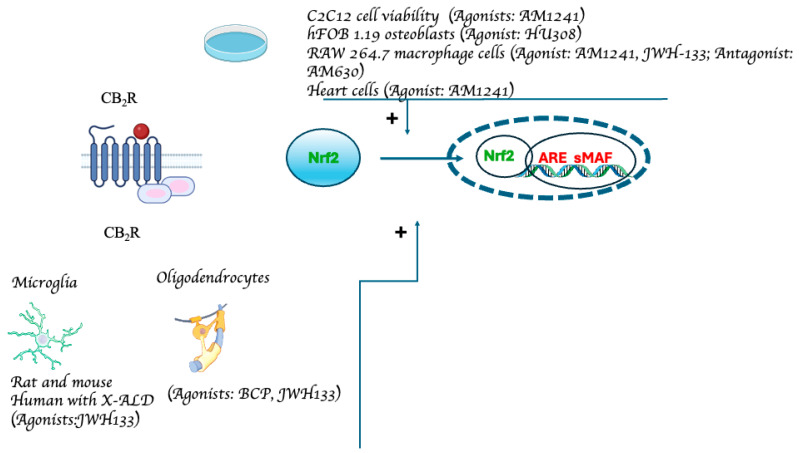
Experimental models demonstrating CB2R-mediated Nfr2 pathway activation. The data from studies investigating the relationship between Nrf2 and cannabinoids via CB_2_R receptors are compiled in the figure. The receptor’s structure and the Nrf2 pathway have been simplified and schematized to aid in the understanding of the figure (from: [48,52,60,61,77,79,80,81]).

This literature review clearly highlights the antioxidant properties of pCBs and eCBs through their interaction with the Nrf2 pathway. However, we are still at a very early stage in the understanding of the pharmacological properties of cannabinoids in this area. Numerous genes that control homeostatic processes within the CNS, such as inflammation, naturally occurring redox metabolism, xenobiotic and carbohydrate metabolism, are enhanced by Nrf2, a transcription factor with crucial functions in defending neurons and glia against harmful insults [88,89]. Research findings show that both pCBs and eCBs can indeed trigger Nrf2 activation at different levels in different neuronal cells, with both micro and macroglia having been reported as the main cells where such interactions mostly occurred. Although pCBs and eCBs seem to be generally able to activate the Nrf2 pathway, further information is still needed to fully understand the modulatory mechanisms and the potential clinical applications of these compounds. In addition, the mechanism by which CB_1_R and CB_2_R stimulation can lead to Nrf2 activation (which under certain circumstances seems to be non-receptor mediated) is not fully understood yet. While the role of CB_2_R in activating the Nfr2 pathway has been established in peripheral tissues, the small number of CNS-focused experiments makes it difficult to fully elucidate the role of this receptor within the CNS. Convincing results of the ability of CB_2_R to activate the Nfr2 pathway have only been obtained from microglia and oligodendrocytes (Figure 2). Evidence that the pCD CBD may interact with the Nrf2 activation at various levels, including Keap1, different kinases, such as p38 mitogen-activated protein kinase, extracellular response kinase (ERK), c-Jun N-terminal kinase (JNK), and AKT, clearly highlight the complex network of signaling pathways activated by the compound. Moreover, by favoring Nrf2 activity, CBD could also reduce Bach1 expression (Nrf2 repressor), increase both p62 (Keap1 repressor) and sirtuin1 (SIRT1, Nrf2 activator) expression, as well as reduce glycogen synthase kinase-3 (GSK3, Nrf2 phosphorylation) [90]. Additional research is needed to determine the exact role played by eCBs at regulating Nrf2 activity within the brain. The possibility to regulate eCBs levels could represent a new potential therapeutic approach to tackle oxidative stress in CNS.

## 8. Literature Search Methods

For this review, we used data from peer-reviewed publications that were indexed in PubMed through 30 March 2024. For assessment and incorporation into this manuscript, only peer-reviewed original research articles and reviews in the English language were assessed.

## Figures and Tables

**Table 1:** The in vivo and in vitro models used to investigate the effects of pCBs and eCBs on Nrf2 activation in the central nervous system are summarized in this table. These studies have mostly investigated the effect of the pCB CBD, which has been shown to modulate the Nrf2 pathway activation in different ways.

Cannabinoids	In Vitro Model	In Vivo Model	References
CBD	BV-2 cells (gene stimulation associated with Nrf2/Hmox1 axis and ARE-Nrf2/ATF4 system)	Mouse model of experimental autoimmune encephalomyelitis (increased the expression of Nrf2 target genes)	[56,57,58]
	MOG35-55-specific T cell line (TMOG) (increased the expression of Nrf2 target genes)		[55,64]
	HT-22 cells (activating the Nrf2-mediated antioxidant response)		
THC	BV-2 cells (gene stimulation associated with Nrf2/Hmox1 axis and ARE-Nrf2/ATF4 system)		[41,57]
	Mice parietal cortex and Hippocampus (Nrf2/Keap1 ratio increase)		
BCP	Oligodendrocytes (OLN-93) (Protective effect through the regulation of Nrf2/HO-1/antiodant axis mediated by CB_2_R)		[52]
CBG	LPS-stimulated RAW 264.7 macrophages on NSC-34 motor neurons (neuroprotective effects increasing Nrf-2 levels)		[53,54]
eCBs	Neurotoxic model of primary hippocampal neurons (neuroprotective effects increasing Nrf-2 levels)		[65]

**Table 2:** The in vitro and in vivo models used to assess the effects of pCBs and eCBs on peripheral Nrf2 activation are summarized in the table. The only pCB investigated in these models was CBD. The compound has shown the ability to activate the Nrf2 pathway in various ways. Moreover, this pathway can be activated by eCBs.

Cannabinoids	In Vitro Model	In Vivo Model	References
CBD	Model of bladder pain syndrome and interstitial cystitis (Nrf2 enhancement)	Mice model of psoriasiform skin lesions (decreasing in plaque and epidermal thickness through CB_2_R activation and higher levels of Nrf2 and HO-1 protein expression	[66,67,68,69,71,72]
	Mouse hepatocytes and L-02 cells exposed to α-amanitin (Nrf2 enhancement)		
	5-fluorouracil-induced oral mucositis in mice and human oral keratinocytes (Nrf2 transcription increase; decrease in Keap1 activation)		
	Keratinocytes exposed to UVA and UVB radiation (activation of Nrf2 and suppression of NFκB transcription factors)	C57BL/6 male mice trained in rigorous exercise (protective effect against myocardial injury via Keap1/Nrf2/HO-1 pathway activation)	
	Primary and immortalized human keratinocytes (HaCaT cell line) (induction of expression of selected Nrf2 target genes)		
eCBs	Breast cancer models (MCF-7 and MDA-MB-231 cell lines) (AEA and inhibition of FAAH activated Nrf2, resulting in HO-1 induction)		[85]

## Data Availability

Data sharing is not applicable to this article as no new data were created or analyzed in this study.
